# The Value of Countryside Elements in the Conservation of a Threatened Arboreal Marsupial *Petaurus norfolcensis* in Agricultural Landscapes of South-Eastern Australia—The Disproportional Value of Scattered Trees

**DOI:** 10.1371/journal.pone.0107178

**Published:** 2014-09-12

**Authors:** Mason J. Crane, David B. Lindenmayer, Ross B. Cunningham

**Affiliations:** Fenner School of Environment and Society, The Australian National University, Canberra, ACT, Australia; University of New South Wales, Australia

## Abstract

Human activities, particularly agriculture, have transformed much of the world's terrestrial environment. Within these anthropogenic landscapes, a variety of relictual and semi-natural habitats exist, which we term countryside elements. The habitat value of countryside elements (hereafter termed ‘elements’) is increasingly recognised. We quantify the relative value of four kinds of such ‘elements’ (*linear roadside remnants*, *native vegetation patches*, *scattered trees* and *tree plantings*) used by a threatened Australian arboreal marsupial, the squirrel glider (*Petaurus norfolcensis*). We examined relationships between home range size and the availability of each ‘element’ and whether the usage was relative to predicted levels of use. The use of ‘elements’ by gliders was largely explained by their availability, but there was a preference for *native vegetation patches* and *scattered trees*. We found home range size was significantly smaller with increasing area of *scattered trees* and a contrasting effect with increasing area of *linear roadside remnants* or *native vegetation patches*. Our work showed that each ‘element’ was used and as such had a role in the conservation of the squirrel glider, but their relative value varied. We illustrate the need to assess the conservation value of countryside elements so they can be incorporated into the holistic management of agricultural landscapes. This work demonstrates the disproportional value of *scattered trees*, underscoring the need to specifically incorporate and/or enhance the protection and recruitment of *scattered trees* in biodiversity conservation policy and management.

## Introduction

Humans have transformed most of the earth's terrestrial biosphere into highly modified biomes [1], resulting in the loss and decline of many species [2]. To counter this, much focus has been on the establishment of formal conservation reserves. While these are a critical component of biodiversity conservation worldwide, reserve networks rarely provide comprehensive, adequate and representative coverage of ecosystems [3]–[6]. It is often the case that those natural ecosystems associated with agricultural land are the most poorly represented in conservation reserve networks. Australian box gum woodlands are a classic example of this [7]. Despite comparatively low levels of reservation in agricultural landscapes, many species associated with such ecosystems still persist there. Such species have been shown to use the relictual and semi-natural habitats of these landscapes (e.g. [8], [9]–[15]). These habitats or countryside elements (‘elements’) [16] often have very different and distinct characteristics, each offering a different range of resources for wildlife.

The role that countryside elements and matrix habitats play in biodiversity conservation is increasingly appreciated. This has been demonstrated by work conducted in Australia [16], Central and South America [10], [17], [18] and West Africa [19]. However, to date there have been few works comparing different kinds of ‘elements’ and quantifying their relative usage for a single species. In this study, we examined the relative value of four kinds of wooded countryside elements in the conservation of a threatened arboreal marsupial, the squirrel glider *Petaurus norfolcensis*, in agricultural landscapes of south-eastern Australia. These ‘elements’ were: *scattered trees*, *tree plantings*, *linear roadside remnants* and small *native vegetation patches*. These four broad kinds of ‘elements’ can be found in many agricultural landscapes around the world (e.g. [20], [21]–[24]). While their use as wildlife habitat has been reported (e.g. [12], [20], [21], [25]–[28]), there are few unequivocal examples of their relative value in the conservation of different species or taxa [16], [29]. To achieve this we address the following questions:

1. Is the use of an ‘element’ for denning and feeding proportional to their availability? If not, is there evidence of preferential usage of some kinds of ‘elements’?
2. Does home range size depend on the proportional availability of an ‘element’? Differences in home range size within species have often been attributed to habitat quality [30]–[32], with smaller home range indicating better quality habitat. If an ‘element’ offers a significantly higher or lower quality habitat relative to others, it would be expected that their availability would have a significant influence on home range size.

Our work is the first to attempt to quantify the relative contribution of different countryside elements for the squirrel glider and is one of the few that attempt to compare the ecological value of countryside elements, using empirical data. An understanding of the value of such ‘elements’ for biodiversity is essential for better integration of biodiversity conservation and broader management of agricultural landscapes. Without such data, managers are in danger of undervaluing certain ‘elements’. This may, in turn, lead to the loss of critical habitats and further threaten associated species in these landscapes.

## Methods

### The squirrel glider

The squirrel glider is a nocturnal, arboreal, gliding marsupial in the Family Petauridae. It is a medium-sized possum weighing between 190–300 g and which feeds on invertebrates, insect exudates, sap of trees and shrubs, and pollen and nectar [33]–[35]. The species is listed as threatened in three of the four Australian states in which it is found (Victoria – *Flora and Fauna Guarantee Act 1988*; South Australia – *National Parks and Wildlife Act 1972*; New South Wales – *Threatened Species Conservation Act 1995*; and Queensland – no formal listing).

### Study area

**Figure 1:**
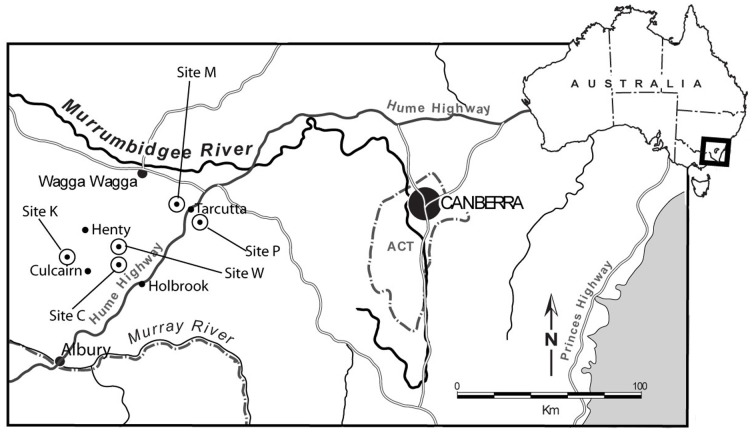
Location of the five study areas (C, K, M, P and W) used in radio-tracking the Squirrel Glider.

Our investigation encompassed five study areas within the south-west slopes of New South Wales, Australia [36] (Figure 1). The region is the most extensively and intensively disturbed of the 13 botanical regions of NSW, with an estimated 85% of the original cover of native vegetation removed in the past 200 years [37]. The five study areas were located in heavily modified agricultural landscapes, used predominantly for livestock grazing and dryland cropping. Study areas were approximately 3 km×3 km. Woody vegetation occurred primarily as relictual scattered paddock trees, native vegetation plantings and remnant temperate *Eucalyptus* woodlands on private lands, road reserves and travelling stock reserves.

### Ethics statement

We conducted trapping and radio tracking under The Australian National University Animal Ethics Committee protocol number C.RE.39.05. Squirrel Gliders are a native species and therefore protected. Relevant permits to handle the animals were obtained from New South Wales Government agencies. Animals were captured in wire mesh cage traps covered with non-transparent, heavy-duty plastic sleeves to minimise stress on animals and protect from cold and wet weather. Qualified wildlife veterinarians anaesthetised captured animals using isofluorane gas delivered via a portable gas anaesthesia machine. Isofluorane anaesthesia ensures recovery of animals within minutes, which is considerably faster than injectable agents, thus minimising holding time and stress. Anaesthetising animals ensured that accurate measurements of body size and reproductive status could be made without undue stress to the animals. It also enabled the veterinarians to properly fit radio-collars and microchip each animal. Gliders not fitted with collars were ear tagged (hamster ear tags, Sieper & Co., Sydney, Australia) in each ear. When animals had recovered from the anaesthetic, they were released at the exact point of capture.

Land accessed was a mix of privately owned farmland, local government managed road reserves, and travelling stock reserves managed by the Livestock Health and Pest Authority and relevant access permissions were obtained from the respective land managers.

### Radio-tracking

We captured gliders using drop-door, wire mesh cage traps (170 mm×200 mm×500 mm) over a three night period at each site in March 2005 [38]. We fitted 32 gliders with a single stage brass loop radio transmitter, weighing 4.5 grams (Sirtrack, New Zealand). When selecting which gliders were to be collared, we preferred adult gliders and attempted to achieve an equal sex ratio and an equal spatial coverage of animals within and between sites. We tracked gliders to their diurnal denning site at least twice a week and to a nocturnal location at least 1–3 times every 14 days, over a 4–5 month period [38], [39]. For each fix, we recorded the countryside element in which the glider was located.

### Home range

We derived home range estimates by using both diurnal and nocturnal fixes. The woody vegetation was scattered or represented in irregularly shaped patches, surrounded by cleared agricultural matrix. Common parametric approaches such as the minimum convex polygon method were therefore unsuitable. This is because they would have (inappropriately) included large areas of unused cleared agricultural land – as has been found by Martin et al. [40] and van der Ree and Bennett [15]. We estimated home range by using the non-parametric, grid cell method [41]. The size of the grid cell is arbitrary and can have a major influence on either underestimating or over-estimating the den range size [41]. Three sized cells were tested 40×40 m, 50×50 m and 60×60 m and compared to the minimum convex polygon (MCP) method (where MCP could be appropriately used, i.e. for some animals in areas dominated by remnant vegetation patches or large clusters of scattered trees). We calculated the minimum convex polygon using Home Range extension within Arc View GIS (ESRI, California, USA). We selected a 50×50 m grid cell as it aligned more closely with the commonly used MCP method. We connected disjointed cells by including cells that were crossed by the most direct line joining to consecutive locations, taking into consideration gap-crossing ability ([i.e. gaps in canopy <70 m; see [42]). We estimated the home range size of each squirrel glider on 95% of fixes. This was done to give an objective, repeatable method of comparison of normal home range [41]. We deleted 5% of fixes at the extremities of each animal's range to reduce the influence of exploratory movements or outlying fixes outside the ‘normal’ home range [15].

### Countryside elements

Within our study area, we recognised four categories of countryside elements that contained woody vegetation.

#### (1) Linear roadside remnants

These were linear strips of remnant vegetation along roads. Remnant roadside vegetation is a major feature across agricultural landscapes providing a network of remnant vegetation corridors across what are otherwise generally heavily cleared landscapes. The width of the roadside reserves in this study ranged from 40–60 m. Vegetation along road reserves is subject to high levels of disturbance such as road construction and maintenance. However, grazing pressure by domestic livestock is often low and irregular. These areas regularly contain regeneration of overstorey species. Native understorey species are generally present but their dominance and diversity varies.

#### (2) Native vegetation patches

These were patches of remnant vegetation where the understorey was dominated by a diverse array of native plants. In this study, these areas were mostly on travelling stock reserves, and some patches of remnant native vegetation on freehold land. The travelling stock route network was established more than 150 years ago to facilitate the movement of domestic livestock between properties and to markets [43]. The network is made up of travelling stock routes (which today are often incorporated into the road reserves) and holding paddocks which are generally referred to as travelling stock reserves (TSRs). Because of their reservation for these purposes, these areas generally escaped clearing and continuous high-intensity livestock grazing. The *native vegetation patches* varied in size with the largest patches occurring on TSRs (≈100 ha) and the smallest patches on freehold land (>5 ha).

#### (3) Scattered trees

These were scattered, (mostly large, old) relictual trees remaining on land used for grazing or cropping. They include dead and living trees, are often widely spaced (more widely spaced than expected in their natural state) and contain a simple understorey, generally dominated by introduced grasses and forbs, with a low diversity of native plant species.

#### (4) Tree plantings

These were Australian native vegetation plantings, generally containing dense stands of trees (predominately *Eucalyptus*) and shrubs (such as *Acacia* and *Melaleuca*). The species composition was predominantly locally indigenous species, but often included species naturally found outside the region. *Tree plantings* vary in their shape and size as they were planted for various purposes such as shelterbelts or to reduce rising water tables [44]. The level of grazing by livestock within them also varied.

The vegetation was assigned to the countryside element of best fit, using the above descriptions as a guide. Where elements were adjacent to each other, boundaries were defined by differences in vegetation structure and composition, and/or management practices. We calculated the area of each countryside element available to an individual squirrel glider by measuring the total area of woody vegetation attributed to that ‘element’, within a 1000 m radius of the centre point of all fixes for each individual glider. We used a 1000 m radius, as 2000 m is approximately the maximum home range length that has been reported for our study species [15]. We measured the area of woody vegetation using geographical information systems software (ArcGIS 9.2-esri) to draw polygons over the canopy of woody vegetation interpreted from satellite imagery (spot 5-Astrium). We deemed that woody vegetation isolated by a gap distance of greater than 70 metres was unavailable to gliders [42].

### Data analyses

For each squirrel glider, our data consisted of a count of the number of fixes in each ‘element’, classified by circadian time - day or night. Associated data were the total area of woody vegetation in each ‘element’, available within a 1000 m radius of the centre point of all fixes for each individual glider. From these data, we can obtain the expected number of fixes by apportioning total fixes according to the relative area of each ‘element’. Thus, for day and night data separately, we have a 2-way contingency table cross-classified by animal ID and ‘element’ type, where the cells are the observed count of the number of fixes and a concomitant variable is the expected number of fixes based on the relative availability of each of the ‘elements’.

### The Model

Considered as a two-way contingency table, our data can be modelled by
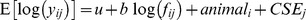
where *y_ij_* are the expected frequencies, *u* is the grand mean, *f_ij_* are the ‘expected’ frequencies derived from the percentage are occupied by *‘element’_j_ animal_i_* and *‘element’_j_* are constants to account for the marginal distribution of the two-way animal by ‘element’ table.

The above model is a particular case of a class of models commonly known as log-linear models, and which are often used in the analyses of multiway contingency tables. The log-linear model belongs to the class of generalised linear models [45]. These models can be fitted by the use of maximum likelihood methods available in many statistical software packages, such as GENSTAT Version 15. The goodness-of-fit of the model and the significance of individual terms can be assessed by examining an analysis of deviance.

We completed further linear regression analysis to quantify relationships between home range size and total woody vegetation and each of the countryside elements. We avoided statistical issues associated with intrinsic collinearity of countryside elements by considering each of the ‘elements’ separately.

## Results

### Radio-tracking

We captured 52 individual gliders and fitted 32 with radio-transmitting collars (see Table 1). The numbers of fixes for individuals varied as signals were lost for some animals throughout the study. Over a five month period, we tracked gliders to 1027 independent locations (655 diurnal and 372 nocturnal locations). We tracked individual gliders to an average of 21±1.16 (mean ± s.e.) diurnal locations and to 12±0.6 (mean ± s.e.) nocturnal locations.

**Table 1:** Number of squirrel gliders captured/collared at each site.

	Site C	Site K	Site M	Site P	Site W	Total
No. captures	5	8	25	3	11	52
No. collars fitted	5	4	12	3	8	32

### Usage of countryside elements

**Figure 2:**
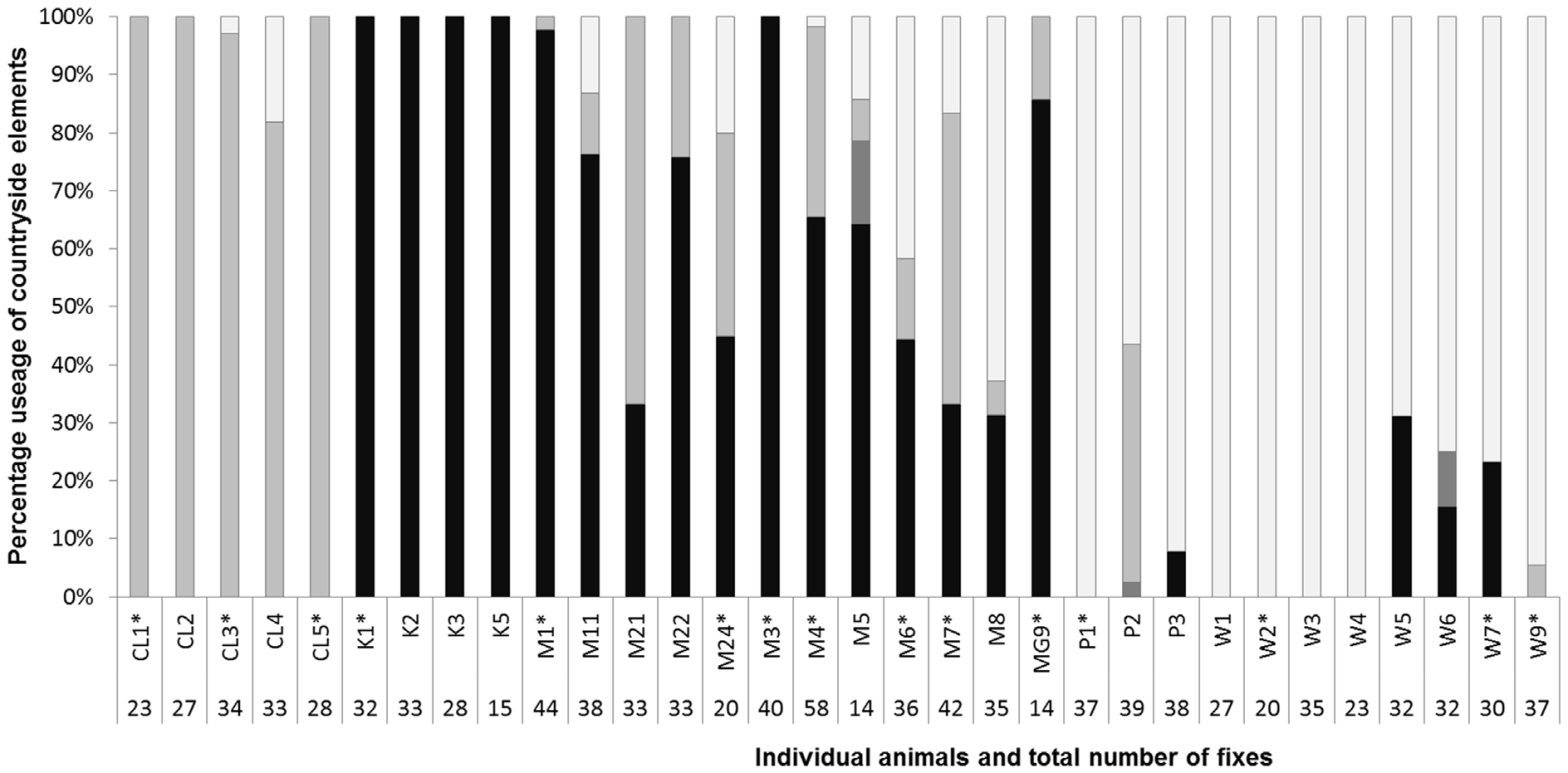
The percentage usage of countryside elements by individual squirrel gliders (* = female). *Remnant vegetation patches* (black shading), *tree planting* (dark grey shading), *linear roadside remnant* (intermediate grey shading) and *scattered trees* (light grey shading).

We found that gliders used all four wooded countryside elements nocturnally and all but one element (*tree plantings*) for diurnal denning. Some individual gliders exclusively used one category of ‘element’, *scattered trees*, *linear roadside remnants* or *native vegetation* (Figure 2).

The fit of the model as defined above is summarised by the analysis of deviance (Table 2). As can be seen from Table 2, 64% (367/576) and 71% of the residual deviance was accounted for by the ‘expected’ count based on the percentage of available ‘elements’ for each animal, for night and day use respectively. The final residual deviance, based on 92 degrees of freedom after fitting the terms for animal, ‘element’ and expected frequency was 209 and 286, for night and day, respectively. This suggested that there remains non-random unexplained variation.

**Table 2:** Accumulated analysis of deviance Table after fitting a sequence of log-linear Poisson models (see section ‘The Model’).

Term	d.f.	Night use	Day use
+ Animal	31	27	76
+ CSE	3	186	391
Residual	91	576	949
+ log(f)	1	367	675
Residual (final)	92	209	286
Total	127	789	1416

**Figure 3:**
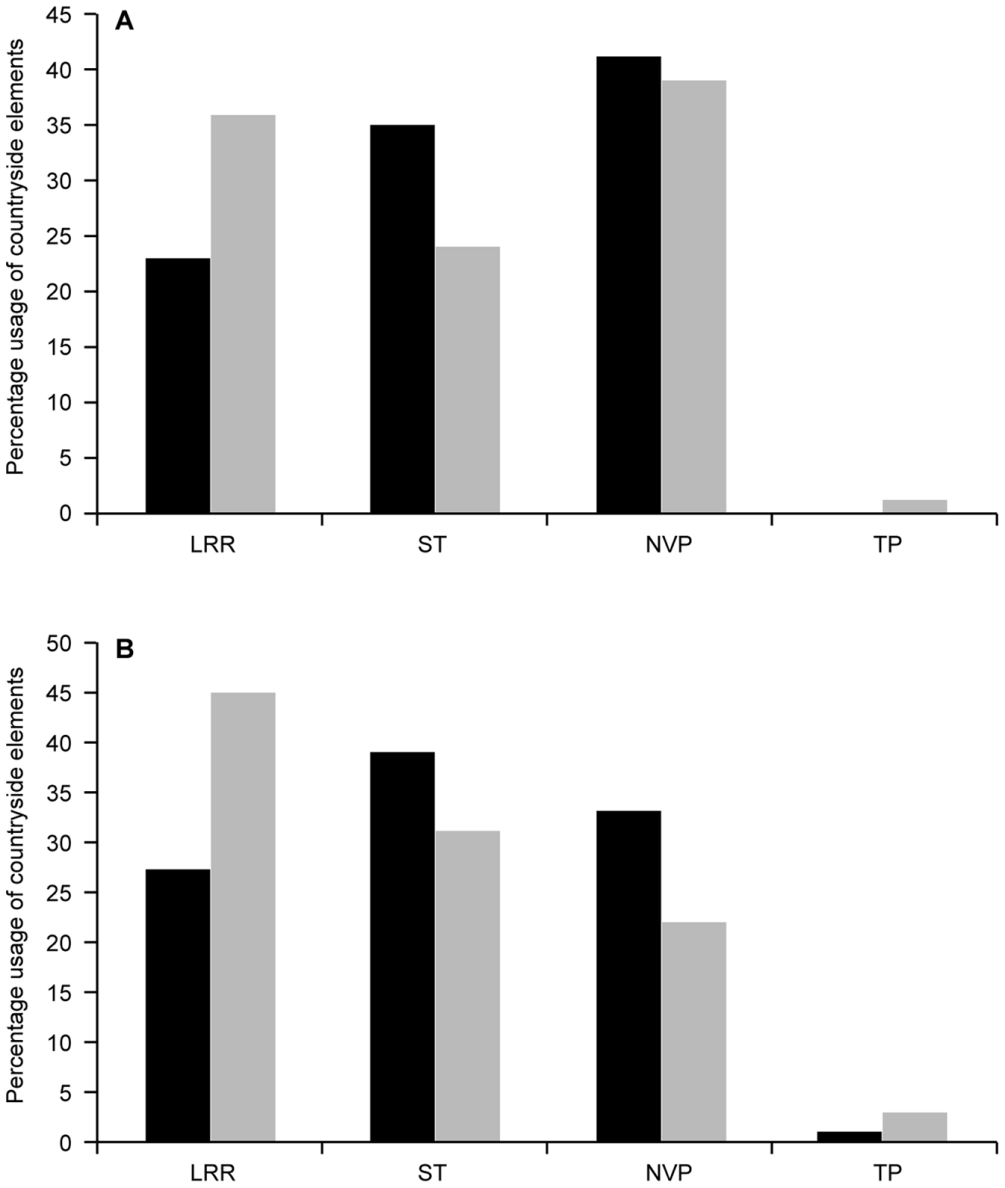
Actual usage verse the predicted usage of countryside elements. LRR  =  Linear roadside remnants, ST  =  Scattered trees, NVP  =  Native vegetation patches, TP  =  Tree plantings. Actual percentage usage is shown as black shading and the predicted percentage usage is shown as grey shading. Panel (a) is based on diurnal fixes, and Panel (b) is based on nocturnal fixes.

A further breakdown of the components of this residual variation revealed evidence of preferential selection of *native vegetation patches* and *scattered trees* nocturnally, and a strong preference to *scattered tree*s for diurnal use, as is shown in Figure 3.

### Relationship between home range size and availability of CSEs

**Figure 4:**
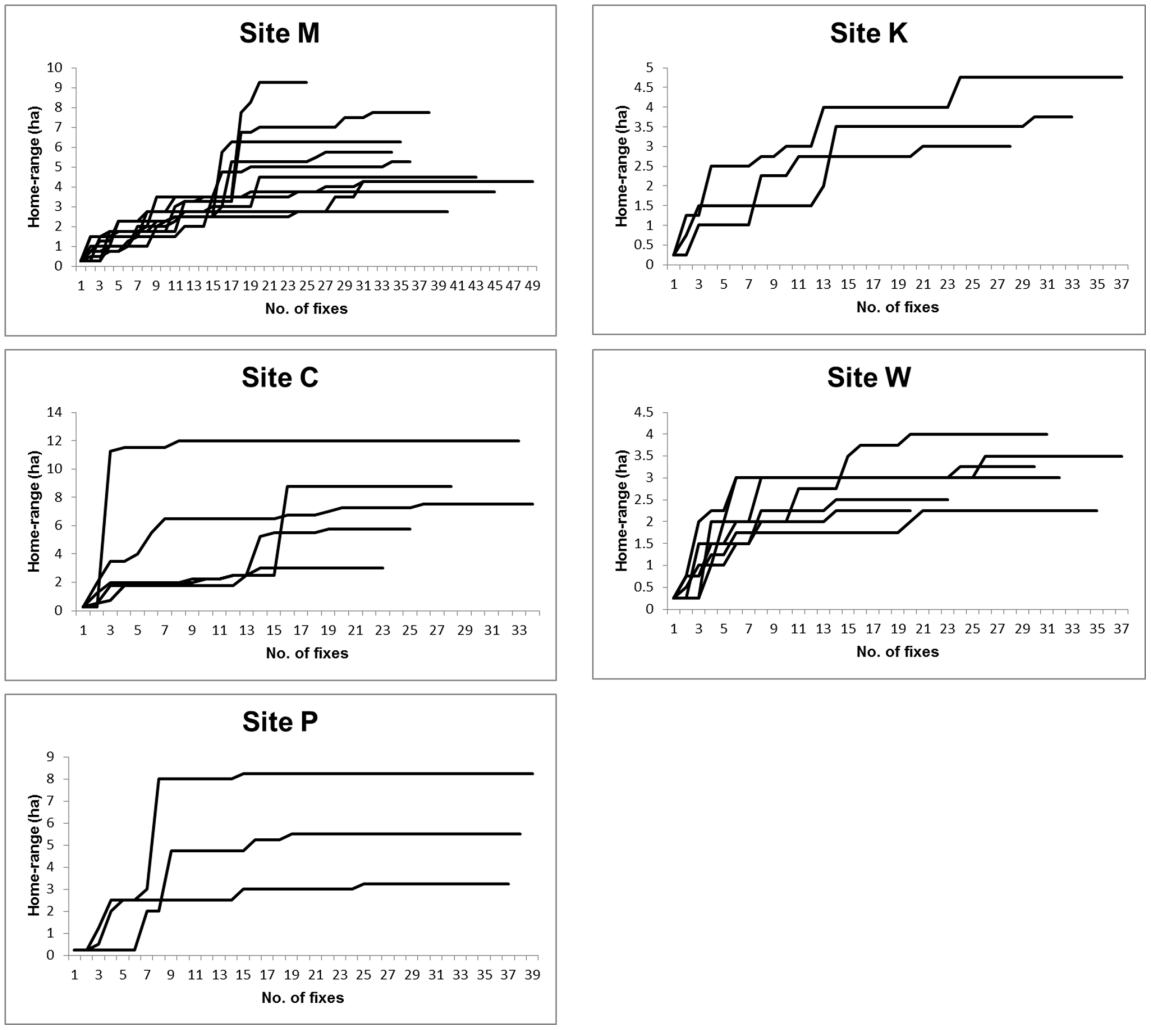
Home range estimates plotted against the number of fixes in sequential tracking order. The fixes are for all animals (with >20 fixes) at each of the five study sites.

Home ranges varied from 2.5 to 12 ha and averaged 4.9±0.45 ha (mean ±1 s.e.). Home range size increased with addition fixes, but plateaued towards the end of our study (Figure 4).

**Figure 5:**
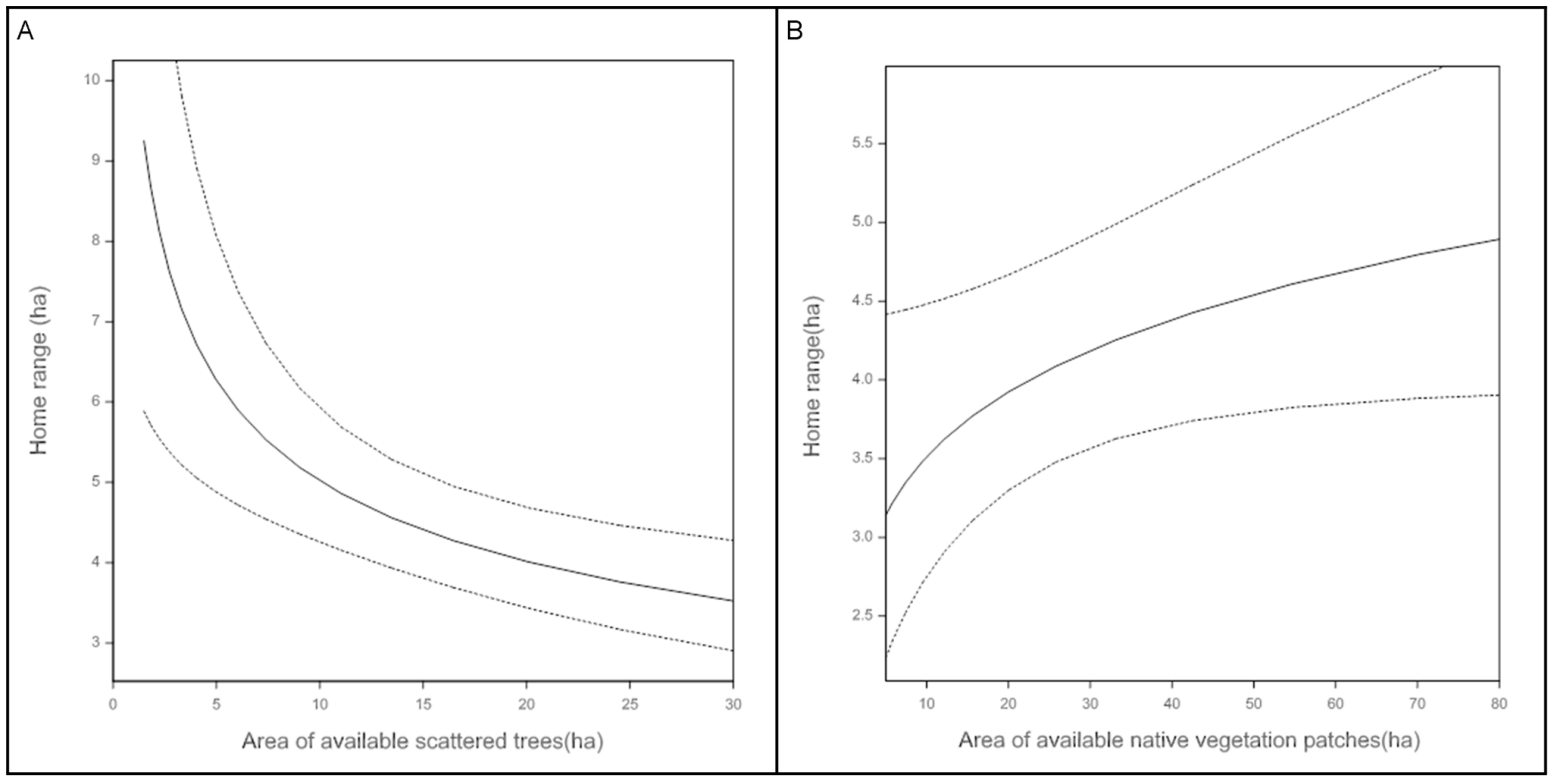
Influence of available area of three countryside elements on squirrel glider home range size. Panel (a) scattered trees, Panel (b) native vegetation patches. Based on 95% of fixes and associated 95% confidence intervals.

We found no significant relationship between total woody vegetation cover and home range size. However, there were significant relationships between home range size and area of woody vegetation within three ‘elements’. We found a significant (p<0.001) negative relationship between home range size and the available area of *scattered trees* and significant (p = 0.009 and p = 0.022) positive relationships with the available area of *native vegetation patches* (Figure 5) and *linear roadside remnants*.

## Discussion

The contribution that countryside elements make towards biodiversity conservation is increasingly recognised in many ecosystems around the world [16]–[19]. The challenge for conservationists is to recognise different ‘elements’ and to understand the role each play in the conservation of different species. Our work is one of the few studies that quantify the relative value of different countryside elements for a single species. We provide empirical evidence of the disproportionate value of *scattered trees* in agricultural landscapes. Our work also highlights the need to examine the roles and values of countryside elements in biodiversity conservation, particular for species such as the squirrel glider which has a distribution largely confined to highly modified agricultural landscapes.

Across our five study sites, gliders relied entirely on countryside elements (*scattered trees*, *linear remnant vegetation*, *native vegetation patches* and *tree plantings*) located within road reserves, traveling stock reserves and freehold land. Our study demonstrates that the squirrel glider will use all of these four wooded ‘elements’, with availability being a key factor in determining usage.

In agricultural landscapes, roadside vegetation provides important habitat for many species of wildlife, particularly in facilitating migration and dispersal [20], [46]–[48]. We found that the squirrel glider would commonly use *linear roadside remnants*, with some individuals relying entirely on this ‘element’. The ability of *linear roadside remnants* to support squirrel glider populations has been previously reported [28]. While it is clear this kind of countryside element is of conservation importance to this species, we found evidence that suggests *linear roadside remnants* may provide inferior quality habitat compared *to native vegetation patches* and *scattered trees*. Larger home range sizes associated with increased area *of linear roadside remnants* and the under-utilisation of this ‘element’ is evidence of this. This may be explained by structural differences in the vegetation between the ‘elements’, such as a *linear roadside remnants* containing a higher proportion of small regrowth trees (which have been shown to offer poorer quality habitat than large trees [34], [38], [39]). Issues associated with the geometry of linear habitats also may explain our findings. Lindenmayer et al. [49] and Recher et al. [50] highlight the problems for species inhabiting linear habitats, such as disrupted social behaviour and additional expenditure of energy in obtaining food.

While *native vegetation patches* in agricultural landscapes are often small and highly fragmented, they have been shown to be important habitat for many species [24], [51], [52]. In our study, the majority (over 90%) of *native vegetation patches* occurred on traveling stock reserves. These reserves themselves have been shown to have high conservation value (higher than remnant vegetation on private land, particularly for arboreal marsupials) [53]. We found that *native vegetation patches* were heavily used by gliders, with a number of individuals using this ‘element’ exclusively. The use of *native vegetation patches* was higher than predicted for both diurnal and nocturnal activity. While *native vegetation patches* were preferentially used, gliders in areas dominated by *native vegetation patches* had larger home ranges. This result may suggest that while quality habitat exists within *native vegetation patches*, it is often dispersed among poorer habitats, resulting in gliders having to cover larger areas to gather resources.

*Tree plantings* have been shown to play an important role in the conservation of some species in agricultural landscapes, particularly birds [9], [27]. For arboreal marsupial conservation, *tree plantings* may have a more limited role, at least in the short to medium term [54]. Our data indicated that *tree plantings* were used less than predicted based on availability. When compared to other treed ‘elements’, plantings would seem to be of minor importance to the species. However, *tree plantings* were nevertheless still used and should not be discounted as their value may increase over time as trees mature, given squirrel gliders’ preference for large trees [34], [38], [39]. *Tree plantings* are the only countryside element examined here that can be readily introduced into these highly modified landscape. Our data suggests that for *tree plantings* to be used, they must be located in association with other countryside elements containing remnant trees.

*Scattered trees* are widely recognised as a critically important countryside element in many agricultural landscapes globally and are considered a keystone structure in such landscapes [23]. Numerous studies have highlighted the ecological value of *scattered trees* for various taxa of wildlife [12], [16], [25], [26], [52]. Fischer et al. [29] have shown that *scattered trees* have a disproportionate value for birds and bats. Our work provides further evidence of this. The home ranges of gliders were significantly smaller with increasing coverage of *scattered trees*, an opposite pattern to all other ‘elements’. We also found that *scattered trees* were used at higher rates than predicted, particularly as diurnal den sites. Both results indicate that scattered trees have a comparatively higher habitat value for the squirrel glider than the other three kinds of countryside elements we examined.

*Scattered trees* are often the oldest living structures in disturbed landscapes [23]. This was the case in our study, with *scattered trees* represented by predominately large mature relictual *Eucalyptus* trees. These trees generally had well developed hollows and a large canopy, both kinds of resources have been shown to be preferentially sought by the squirrel glider [34], [38], [39]. Despite this, *scattered trees* are arguably under the greatest threat of all countryside elements and populations of such trees are rapidly diminishing resource in many agricultural landscapes [55], [56]. It is estimated that tens of millions of *scattered trees* will be lost in grazed landscapes in Australia over the next 50 years, due to factors such as natural attrition, clearing, and a lack of regeneration [55] – a problem common to many agricultural landscapes globally [56]. Current conservation strategies offer little protection for *scattered trees* [55], and they are cleared for cropping and irrigation [57]. Without a concerted shift in conservation policy and awareness in general of the value of *scattered trees*, there is a real risk of losing this critical countryside element.

## Conclusions

Agricultural landscapes are dynamic. Changing technologies, land uses and attitudes continually transform these systems [58]. There are significant threats to the integrity and extent of various kinds of countryside elements within agricultural landscapes. Therefore, understanding the contribution they make to biodiversity conservation is essential. In the case of the squirrel glider, we found that four kinds of countryside elements are important, but their relative values vary. We suggest that these countryside elements need to be better integrated into landscape management.
